# Hemocompatibile Thin Films Assessed under Blood Flow Shear Forces

**DOI:** 10.3390/molecules27175696

**Published:** 2022-09-04

**Authors:** Roman Major, Grażyna Wilczek, Justyna Więcek, Maciej Gawlikowski, Hanna Plutecka, Katarzyna Kasperkiewicz, Marcin Kot, Małgorzata Pomorska, Roman Ostrowski, Magdalena Kopernik

**Affiliations:** 1 Institute of Metallurgy and Materials Science, Polish Academy of Sciences, 25 Reymonta St., 30-059 Cracow, Poland; 2 Institute of Biology, Biotechnology and Environmental Protection, University of Silesia, Bankowa St. 9, 40-007 Katowice, Poland; 3 Faculty of Biomedical Engineering, Department of Biosensors and Processing of Biomedical Signals, Silesian University of Technology, Roosevelt St. 40, 41-800 Zabrze, Poland; 4 Division of Molecular Biology and Clinical Genetics, Faculty of Medicine, Jagiellonian University Medical College, Skawinska St. 8, 31-066 Cracow, Poland; 5 Institute of Biology, Biotechnology and Environmental Protection, Faculty of Natural Sciences, University of Silesia in Katowice, Jagiellońska St., 2840-032 Katowice, Poland; 6 Faculty of Mechanical Engineering and Robotics, AGH University of Science and Technology, Al. Mickiewicza 30, 30-059 Cracow, Poland; 7 Institute of Optoelectronics, Military University of Technology, Gen. S. Kaliskiego St. 2, 00-908 Warsaw, Poland; 8 Faculty of Metals Engineering and Industrial Computer Science, AGH University of Science and Technology, Al. Mickiewicza 30, 30-059 Cracow, Poland

**Keywords:** blood–material interaction, thin coatings, radial flow chamber, blood shear stress, nanoindentation, Impact-R, hemocompatibility, heart valve

## Abstract

The aim of this study was to minimize the risk of life-threatening thromboembolism in the ventricle through the use of a new biomimetic heart valve based on metal–polymer composites. Finite volume element simulations of blood adhesion to the material were carried out, encompassing radial flow and the cone and plane test together with determination of the effect of boundary conditions. Both tilt-disc and bicuspid valves do not have optimized blood flow due to their design based on rigid valve materials (leaflet made of pyrolytic carbon). The main objective was the development of materials with specific properties dedicated to contact with blood. Materials were evaluated by dynamic tests using blood, concentrates, and whole human blood. Hemostability tests under hydrodynamic conditions were related to the mechanical properties of thin-film materials obtained from tribological tests. The quality of the coatings was high enough to avoid damage to the coating even as they were exposed up to maximum loading. Analysis towards blood concentrates of the hydrogenated carbon sample and the nitrogen-doped hydrogenated carbon sample revealed that the interaction of the coating with erythrocytes was the strongest. Hemocompatibility evaluation under hydrodynamic conditions confirmed very good properties of the developed coatings.

## 1. Introduction

The heart valve is a regulatory component of the circulatory system that is responsible for the proper blood flow in the heart. The correct operation of the valves ensures an appropriate hemodynamic state of the human being and the correct perfusion of organs, as well as tissue microperfusion. The valves, however, can be destroyed for various reasons [1].

Dysfunction of any of the four heart valves causes heart or bacterial myocarditis. Over time, dysfunction intensifies, increasing heart pain making any exertion dangerous. Sometimes the only chance to save a patient is to replace his/her own valve with an artificial one.

Mechanical valves are preferred in situations where such a prosthesis is acceptable by the patient, i.e., there is neither contraindication for anticoagulant therapy nor a high risk of damage to the valve structure. Therefore, the mechanical valves are considered for the patient who already has other implanted prosthesis requiring anticoagulant therapy due to a high risk of thromboembolic complications. Such implants are also used for the patients requiring chronic anticoagulant therapy when the patient is young and has a long-anticipated survival time [2,3,4].

Implantation of an artificial valve is aimed at improving the patient’s health and is sometimes a life-saving procedure. As an invasive procedure, valve implantation may sometimes involve dangerous complications. Undesirable consequences that are related to prosthetic dysfunction can be divided into two groups: structural (depending on the structure or function of the prosthesis) and unstructured. Generally, disordered valve function may take the form of stenosis, regurgitation, or both at the same time [5,6,7].

Structural abnormalities in a mechanical prosthesis result from degradation of the materials of which it is made. The reason of this phenomenon is either inadequate strength of materials or the effect of cavitation (the formation of microbubbles around the prosthesis, carrying with them a large dose of energy that can damage the elements of the valve) [8,9,10]. There are well documented cases of the formation of cavitation bubbles on HALL valves (manufactured by Medtronic) [11]. In contrast, the reason for the structural dysfunction of a biological prosthesis is usually the process of calcification of the tissues that are used for its construction. For this reason, the average lifetime of biological valves is ~10 years. After this time, in 40% of cases it is necessary to surgically replace the valve [12]. Non-structural complications may include valve-related leakage; depending on the type of valve, the method of implantation and the presence of calcification in the valvular ring may lead to hemolytic anemia, functional narrowing caused by improper valve size selection in relation to the weight and height of the patient. A creation of the pannus, i.e., excessive fibrous tissue, takes place in the healing process, narrowing the light flow, or valve thrombosis. It is related to the type of valve (with a greater risk for a mechanical valve) and may consequently lead to life-threatening peripheral embolism [13]. An important and unfortunately common complication of artificial valve implantation is infective endocarditis. It develops as a result of infection of the membrane lining the inside of the heart cavity. Degraded endocarditis can lead to the formation of abscesses and fistulas on the valve components, and thus to their dysfunction. Sometimes reoperation is necessary in such a case. The proposed metal-reinforced polymer materials in combination with the high precision manufacturing technologies are a novel, innovative concept, offering numerous advantages by combining flexibility with the micromechanical stability on the macro-scale. However, these techniques are presently not used for any blood contact applications [10,14,15,16,17]. Nevertheless, the production of a hybrid composite part, consisting of a fragile metal insert and an ultra-thin-walled polymer cover, is still a challenge, especially given the small dimensions and tolerances of heart valve components. This requires full knowledge of the rheological properties of the polymer (TPU—Thermoplastic Polyurethane) [18,19,20,21,22], simulation methods exceeding the current state-of-the-art practices, precise positioning of the metal frame, and an adapted micro-injection molding cycle to avoid any collapsing of the frame due to the high pressure of the melt during filling in of the cavity [9,10,23,24,25,26,27]. Additionally, some aspects of heart-valve sealing to prevent wear of the TPU leaflet can be challenging. Deposition of thin flexible coatings (doped DLC [28,29,30]) on the TPU (Bionate^®^ II PCU 80A, DSM Biomedical, Exton, PA, USA), molecular weight: 258000 g/mol) and titanium by sputtering techniques may provide low-friction based wear resistance and may additionally improve the hemocompatibility of the TPU. High, biocorrosion-resistant adhesion of such hard coatings on the elastic TPU and controlled biofunctionality require adequate ion plasma pretreatment [31,32,33] and fluoridization or oxidation post-treatments [34,35,36,37,38,39]. The manufacturing of a fragile titanium is inserted with a flexibility that is based on the local mesh-width and thickness requires sophisticated high-precision milling and laser cutting [40,41,42,43,44], vacuum heat treatment to control the proper mechanical properties of the titanium, and surface finishing by electropolishing. The novelty and scientific contribution to the discipline comes from the development of materials in the form of thin coatings characterized by low hemocompatibility for long-term direct contact with blood for the desired application in the form of silent heart valves dedicated to the new generation of cardiac assist chambers. Several materials have been shown to outperform thermoplastic polyurethane in terms of thrombogenicity, coagulation, and hemolysis behavior. However, the literature lacks complete test results for such coatings on soft substrates, yet they are essential for flexible heart valves in terms of high-cycle fatigue resistance.

## 2. Materials and Methods

### 2.1. Surface Modification

The deposition of the coatings was performed by physical and plasma-enhanced chemical vapor deposition (PVD and PECVD, respectively) on a titanium-based substrate and TPU substrate (Bionate^®^ II PCU 80A, DSM Biomedical, Exton, PA, USA), molecular weight: 258,000 g/mol). The tested coatings were applied to round discs with a diameter of 80 mm for tests in radial flow chamber and with a diameter of 14.4 mm for Impact-R tester and for other studies performed in this paper. A detailed description is presented elsewhere [45]. Prior to the deposition, all substrates were cleaned ultrasonically in alcohol and acetone to remove all adhering contaminations. The manipulation of the cleaned substrates was performed under clean room conditions to prevent any dust deposits on the surfaces. After mounting the substrates in the vacuum chamber (Leybold Oerlikon, Cologne, Germany) in vertical position at ~80 mm in front of the magnetron sputtering cathodes. It was evacuated to a 2 × 10^−5^ mbar starting pressure. Prior to the film deposition, a final cleaning and activation by ion etching was executed by applying the Ar-O_2_ plasma from a linear anode layer ion source (ALS 340, Veeco, Fort Collins, CO, USA). Film deposition itself was performed by different techniques: PECVD by applying the above-mentioned anode layer source was used to achieve hydrogenated tetrahedral amorphous carbon coatings (ta-C:H) in acetylene atmosphere with the partial addition of argon. Magnetron sputtering (SPU) from pure pyrolytic carbon target materials (Schunk, Vienna, Austria) occurred in a mixed atmosphere of acetylene and argon to achieve amorphous hydrogenated carbon films (a-C:H). Additional mixing of nitrogen instead of argon to this atmosphere resulted in the deposition of nitrogen-containing, hydrogenated amorphous carbon films (a-C:H:N) (Table 1). All gases that were used had 5 N quality and were delivered by Linde Gas (Graz, Austria). Various deposition durations resulted in different thicknesses (Table 1), and they were the basis of the investigations. The thickness of the thin layers was measured from the cross section of the TEM microstructure. Foils for TEM analysis were prepared using Focused Ion beam.

All the important parameters of the coating deposition are presented in Table 1.

### 2.2. Fundamental Research Material Evaluation

#### 2.2.1. Surface Wettability

The contact angle tests were performed using a goniometer (Möller–Wedel Optical, Wedel, Germany). 1.5 µL of distilled water was automatically spotted onto the sample, and then the contact angle information was collected using the goniometer camera for one minute. The program Surftens 4.5 automatically averaged the collected values. Three separate measurements were made for each sample, each time spotting a drop on the “fresh” surface.

#### 2.2.2. Microstructure Analysis

A Dual-Beam SCIOS II scanning microscope from ThermoFisher (Waltham, MA, USA), equipped with an FEG electron gun (30 kV) and a gallium ion gun (30 kV and max. current 65 nA), was used to study the surface after blood testing. The microscope is equipped with an EasyLift micromanipulator. The desiccated blood cells on the surface were dehydrated in an alcoholic series and dried. To ensure adequate conductivity, Au-Pd coatings of approx. 40 nm were applied to the surface.

#### 2.2.3. Mechanical Evaluation of Thin-Film Materials

The mechanical properties of thin-film materials were tested with the aim to determine hardness and Young’s moduluzs. The indentation tests with the Berkovich or Vickers are the most common used for such assessment [46]. In case of thin-film materials like those in the present experiment, only the former can be used, as these materials have a very high hardness, requiring diamond indenters. The test is based on an analysis of the load/displacement curve during the load/unload cycle.

In the case of thin films, the greatest difficulty is performing the test in such a way that the hardness results are unaffected by the substrate on which the thin film is applied [47,48]. A second very common test is the scratch test, which determines the coating–substrate adhesion. This is of particularly importance because thin-film materials will fulfil their function if they stay attached to the substrate throughout their lifetime. The scratch test using a diamond Rockwell C indenter (Anton Paar, Graz, Austria) is analyzed, relying on characteristic damage forms developed during the interaction of the substrate and the diamond tip subjected to increased loading [49].

#### 2.2.4. Adenosine Triphosphate (ATP) Level and Adenosine Diphosphate/Adenosine Triphosphate (ADP/ATP) Ratio

The aim of this part was to assess fibroblast metabolic condition, in contact with coatings deposited by PVD and PECVD, respectively. Fibroblasts cell line cat. No. C-12302 was used as cells model. The cells in question were freshly isolated from non-tumor tissue. They have a finite life span in vitro. They have neither been genetically modified nor treated and/or selected to produce a continuous cell line. “Normal” cells are primary cells that have been subcultured at least once. The coatings were deposited on a titanium-based substrate. The test allowed us to predict the fate of cells toward proliferation, apoptosis, or necrosis. Assessment of fibroblast degenerative changes was carried out using the ApoSENSOR TM ATP Cell Viability Bioluminescence Assay Kit (BioVision, № K254) and ApoSENSOR ADP/ATP Ratio Bioluminescence Assay Kit (BioVision, № K255. Culture medium was removed and treated adherent cells with Nuclear Releasing Reagent for 5 min at room temperature with gentle shaking. Then, 10 µL of the cultured cells was transfer into luminometer plate, according to the manufacturer’s protocols. Intracellular ATP level is an important determinant of the type of cell death. Mutual relationships between nucleotides: ATP and ADP (ratio ADP/ATP) allow predict fate of cells toward proliferation or death. Culture medium was removed and treated adherent cells with Nuclear Releasing Reagent for 5 min at room temperature with gentle shaking. The ApoSENSOR™ Assay Kits utilizes bioluminescent detection of the ATP levels for a rapid screening of apoptosis and cell proliferation simultaneously in cells. Determination of ATP concentration is based on the reaction of oxidative decarboxylation of luciferin catalyzed by luciferase, in the presence of high energy ATP and magnesium ions. As a result of the reaction, light intensity is proportional to the ATP content in the examined sample. The light intensity is measured at a wavelength of 562 nm. ADP level was measured by its conversion to ATP that is detected using the same reaction. The decrease in ATP concentration and increase in ADP are much more pronounced in necrosis than apoptosis. The absolute ATP amount in samples was calculated on the basis of prepared ATP standard curve (using the ATP standard provided in the kit). Additionally, in all the samples of fibroblasts suspension from the control group and groups exposed to selected coatings, protein content was measured according to Bradford [50] using BSA (Fluka, protein content > 95%) as standard. Results were given in nmol ATP mg^−1^ total protein and for ADP/ATP ratio in unitless values. 

### 2.3. Applied Research Material Evaluation Based on Hemocompatibility Assessed in Dynamic Conditions

#### 2.3.1. Models of Blood–Material Adhesion Tests: Radial Flow and Impact-R

The 3D models of blood–material adhesion tests were developed using finite volume method (FVM) in the Ansys Fluent code (Ansys, Inc., Canonsburg, PA, USA): radial flow test model and cone and plane test (Impact-R) model. The first test as a fundamental research test measures cell detachment which occurs for the critical value of the stress [51,52], and the second as a technology test is designed to examine the influence of shear stresses on cardiovascular tissue [53,54]. The values of shear stress and blood flow velocity were computed, implementing boundary conditions imitating real materials surface roughness.

**Figure 1:**
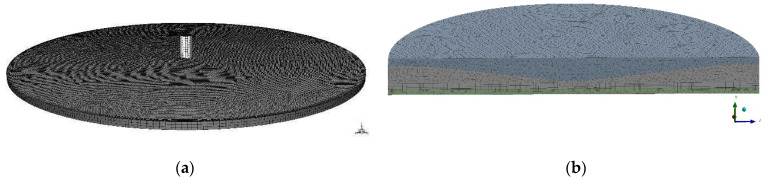
(**a**) FVM meshes of fluid domains of radial flow test; (**b**) FVM meshes of fluid domains of Impact-R test.

The 3D FVM model of the radial flow test was composed of two fluid domains (Figure 1a). The bottom wide cylinder of diameter 14 mm and height 0.25 mm, the area into which the liquid flows into the system, and the upper thinner cylinder, the inflow channel of diameter 0.5 mm and height 0.9 mm. The shape of the radial flow test and the boundary conditions were based on the physical model of the radial flow test developed in [51]. The initial flow rate was equal to 36 mL/min, the properties of the fluid medium-PBS (Phosphate Buffered Saline) [55] are density 1.005584 g/mL and viscosity 0.010219 kg/m.s.

The FVM mesh is composed of 1.5 × 10^5^ finite volume cells and 1.1 × 10^5^ nodes (Figure 1b). The lowest orthogonal quality is equal to 0.8 in the generated mesh, which means that the lowest quality mesh metrics is very good. The lowest skewness mesh metrics are equal to 0.3, which means that the lowest quality of mesh metrics is also very good.

The following solution methods were selected: pressure-velocity coupling solution with scheme of coupled spatial discretization, transient formulation with second order implicit, gradient-least square cell based, pressure-second order, and momentum-second order upwind. The 1 s of simulation was divided into time steps using adaptive time-stepping method. The average computing time of 3.0 × 10^4^ time steps was equal to 5 h using parallel computing on four processors. The standard PC (Intel Core i7, 7400, 3 GHz, 8 GB RAM) was used for all computations in the present study.

The dimensions of the modeled cone and plate test (also called Impact-R) have been taken from the physical model of the test described in [56]. A teflon cone with a radius of 6.5 mm and an angle of 88° rotated in the blood at 720 revolutions per minute for five minutes. The shape of the chamber in which the cone rotated corresponded to a polystyrene cylinder with a radius of 7 mm. The distance between the cone and the cylinder wall was 0.5 mm.

Standard solution initialization computed from inlet and pressure-velocity coupling using coupled scheme of solution were adopted. The simulations were carried out on FVM grids of the cylinder flow domain and cone solid domain with the number of elements equal to 3.4 × 10^6^ and the number of nodes equal to 6.5 × 10^5^ (Figure 1b). The meshes had very good orthogonal quality. The whole simulation of 5 × 10^3^ iterations lasted for several days on PC specified in the present section.

The non-Newtonian blood model and *k*-*ε* turbulence model [57,58] with standard wall functions were applied for radial flow and Impact-R test models. The constants in the *k*-*ε* model are: *C_1ε_* = 1.44, *C_2ε_* = 1.92, *C_μ_* = 0.09, *σ_k_* = 1.0, *σ_ε_* = 1.3. The blood flow has been modeled according to the following power law [58,59]:

(1) $$\eta =k{\dot{\tau }}^{n-1},$$

where *k* is the consistency coefficient and *k* = 0.134 pa/s, *n* is the power term and flow behavior index, *n* = 0.785, and $\dot{\tau }$ is the shear rate.

A stationary wall with no-slip shear condition and roughness parameters [60]: *C*_S_ = 0.5 and *K*_S_ = 10 were applied to the fluid domain represented by the cylinder of Impact-R test and bottom part of radial flow test models. The height of the roughness and its homogeneity were adopted to the experimental observations.

#### 2.3.2. Blood Cell Detachment under Shear Flow

**Figure 2:**
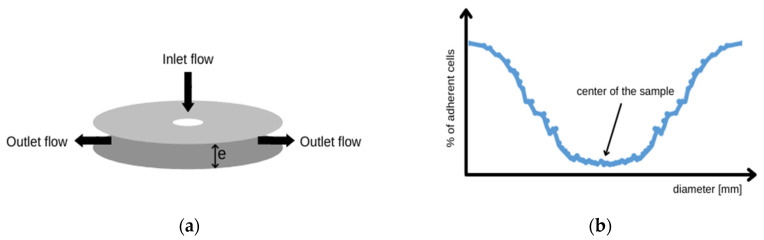

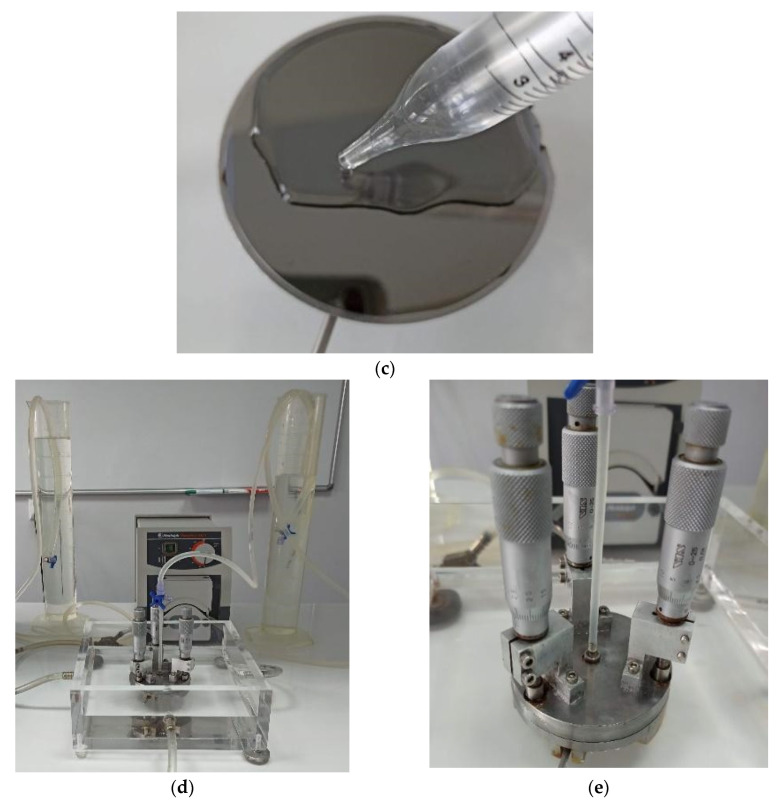
(**a**) Schematic of radial flow chamber; (**b**) Typical graph pattern obtained from results after the radial flow cell test; (**c**) Method of applying the solution of morphotic elements to the sample; (**d**) Experimental setup of radial flow chamber; (**e**) Tripod-flat stainless-steel disc (80 mm diameter) pierced in its center (1.5 mm orifice diameter).

The radial flow chamber was used to assess the kinetics of cell detachment from the test material. It is a dedicated device for performing fundamental cell–substrate interaction assessment. Cells themselves are not self-adhesive objects due to the presence of a repulsive polymer coating called glycocalyx [61,62]. The ability to form specific contacts depends on families of integrin-type adhesion proteins. Many of these adhesion proteins are also receptors, eliciting a cellular response upon ligand binding. Cell signaling results in further cell stabilization [51,63,64]. Quantitative measurement of cell–substrate adhesion is difficult because this process involves the collective behavior of individual proteins encapsulated on a two-dimensional membrane geometry. Therefore, measuring cell–substrate adhesion is intrinsically linked to modelling the geometry of the contact zone. Experimental application of external forces is essential to obtain quantitative information on the adhesive strength and relevant mechanical parameters of living cells. In this case, the model should explain how the mechanical energy associated with the applied forces is dissipated within the cell structure, creating deformation, and at the cell–substrate interface, allowing the cell–substrate interaction to be broken. The radial flow chamber is the second tester available for testing the hemocompatibility of materials under hydrodynamic conditions. This method was used to assess the cell–substrate interaction in the work. The chamber was designed using assumptions from the acute thrombogenicity test [51]. Tension is generated between two discs, the upper one with the inflow hole and the lower one with the circular sample to which the cells adhere (Figure 2a). The stress generated between the two discs depends on two factors. The first is the distance “*e*” between the discs, and the second is the height of the fluid column from which the inflow was fed on a communicating vessel basis [65].

The geometry of the system causes the flow rate to decrease with the distance from the opening in the upper disc from which the inflow is fed. If the value of the hydrodynamic force reaches a critical point, the cell is detached to the surface and entrained into the circulating system. Provided that the cell is treated as an elastic body, it can be assumed that the stress exerted on the cell decreases inversely proportional to the radius “*r*” according to formula (2):

(2) $$\sigma \left(r\right)=\frac{3D\eta }{\pi re^{2}}$$

where *σ* is shear stress, *D* is flow velocity, *η*-dynamic viscosity of the leaching medium, *r*-disc radius (sample radius), *e*-distance between the lower disc, on which the sample is placed, and the upper disc, from which the inflow is supplied.

As cells are washed away from the sample surface, characteristic patterns begin to form on the sample surface, referred to as “washout valleys” shown in Figure 2b. The distance “*e*” was set at 250 µm during the test. The research was carried out with the use of concentrates of red blood cells (erythrocytes) as well as buffy coat (a mixture of thrombocytes and leukocytes). Blood concentrates were purchased from the Regional Blood Donation Centre. One unit of red blood cell concentrate is the blood component obtained from one unit of whole blood after the removal of most plasma. It contains all red blood cells present in one unit of whole blood (hematocrit from 0.65 to 0.75) and, depending on the centrifugation conditions, a different number of platelet cells and leukocytes. The preparation was carried out during one stage, as soon as possible after completion of the donation. The blood cell concentrates were diluted so that the individual cells were visible and that these cells formed the appropriate characteristic elution patterns developed during the experiment (Figure 2b). PBS pH 7.4 was used as a solvent in the experiment. For the concentrate of erythrocytes, a 1: 4000 dilution was prepared in PBS solution and for the buffy coat 1:500 dilution. The final concentration was determined by the Naubauer counting chamber and for RBC it was 14.4 cells/μL (SD 4.4) and for Buffy coat it was 440.0 cells/μL (SD 89.1). The sample was cleaned with 70% ethyl alcohol and distilled water. Then, it was placed on the table, and 14 mL of the previously prepared concentrate of morphotic elements was gently applied (Figure 2c). Cells adhere to the substrate after about 2 min. However, according to the article [51], the time was extended to 10 min. After this time, the inflow started, which, depending on the experiment, lasted 2.5 min, 5 min, or 7.5 min (Figure 2d,e). The flow rate was constant during the entire experiment and depended individually on the tested material. The sample was then imaged with a confocal microscope (Carl Zeiss, LSM 5 EXCITER, Zeiss, Jena, Germany).

#### 2.3.3. A Simplified Method for Assessing Hemocompatibility under High Shear Stresses

The test allows us to evaluate materials under dynamic conditions in vitro as regards hemocompatibility. Instead of a linear flow cell, the test is based on the Cone and Plate Analyzer (CPA), an instrument approved for clinical use in the assessment of thrombotic diseases and the efficacy of antiplatelet drugs. The rationale for choosing this test is usually as follows:

- Lower donor blood requirements for the test—only 130 μL of blood per test,
- Availability of a commercial testing device,
- The accepted principle of a rotating cone and plate as a source of shear stress, as in rheometers or viscometers.

**Figure 3:**
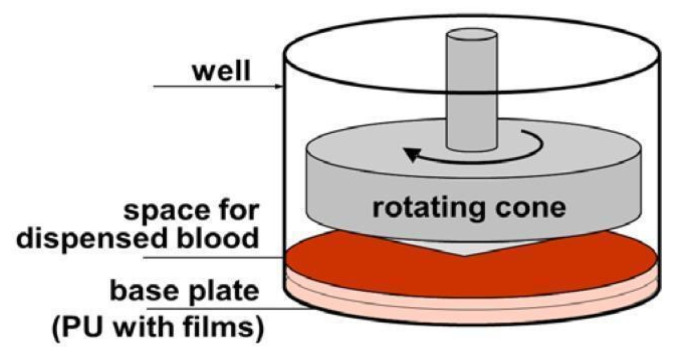
Schematic of Impact-R test [61] (copyright permission has been provided).

This test also allows us to investigate the effect of time (between consecutive days) on the reproducibility of results in a single blood donor. A device under the trade name Impact-R allows platelet function tests to assess platelet adhesion and aggregation under arterial flow conditions. The rotating cone (780 rpm) produces laminar blood flow, which creates uniform shear stresses on the surface. The shear force at each point on the surface is inversely proportional to the distance between the cone and plate and directly proportional to the angular velocity (Figure 3). Due to the special angle (2°) of the cone used in the CPA apparatus, the decrease in the shearing force at the plate surface (caused by the increased distance) is compensated by the increasing angular velocity [65,66,67].

The main research technique after Impact-R test was flow cytometry. Samples were analyzed using EPICS XL flow cytometer (Beckman Coulter Inc., Brea, CA, USA). Expression of platelet activation markers was measured on CD61 gated objects using PAC-1 antibody for conformational change of glycoprotein IIb/IIIa, using CD62P for P-selectin. Integrated fluorescence of the activation marker was calculated as a multiplication total of geometric mean fluorescence by percentage of marker-positive objects. Aggregates of platelets were analyzed after erythrocyte lysis. Scanning electron microscopy was only used as a complementary technique. The results from these tests were only used as a verification of the experiments performed using the cytometric technique.

To assess hemocompatibility, whole human venous blood samples were collected. P-selectin and fibrinogen receptor GP IIb-IIIa (PAC-1) were selected as platelet activation markers. Positive and negative controls were used to determine the number of aggregates. Negative controls were obtained from human blood samples taken on sodium citrate (baseline-Bas) to prevent clotting. Positive controls were prepared by mixing blood with adenosine diphosphate (ADP) to a final concentration of 20 mM. In the hemocompatibility test, polyurethane was chosen as the reference material because it is known to be widely used in cardiac surgery.

## 3. Results

### 3.1. Surface Wetttability

**Figure 4:**
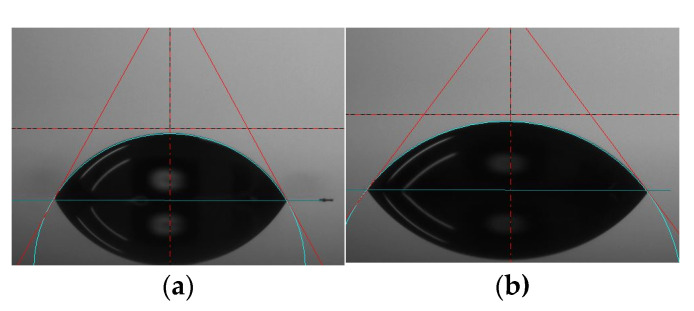

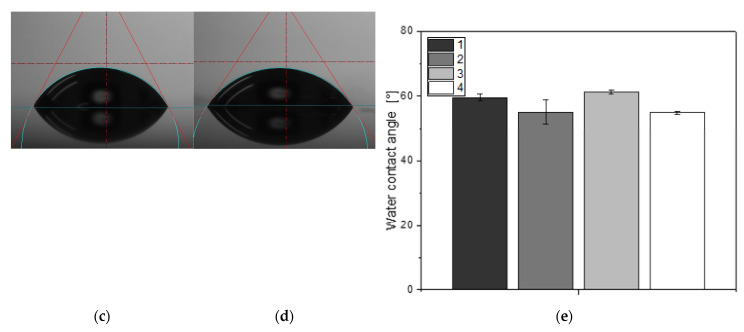
Contact angle measurements. Images of a water droplet on the surfaces on (**a**) coating 1 (according to Table 1), (**b**) coating 2 (according to Table 1), (**c**) coating 3 (according to Table 1), (**d**) coating 4 (according to Table 1), (**e**) Contact angle of the tested coatings.

The water contact angles on the surfaces were shown in Figure 4. All four coatings showed hydrophilic performance (*θ* < 90°). The average water contact angle for coating number 1 (according to Table 1) was 59.52°, for coating 2 it was 55.04°, and for coating 3 it was 61.18°. Coating number 4 generated the most hydrophilic surface with measured contact angles of 54.83°.

### 3.2. Microstructure Analysis

**Figure 5:**
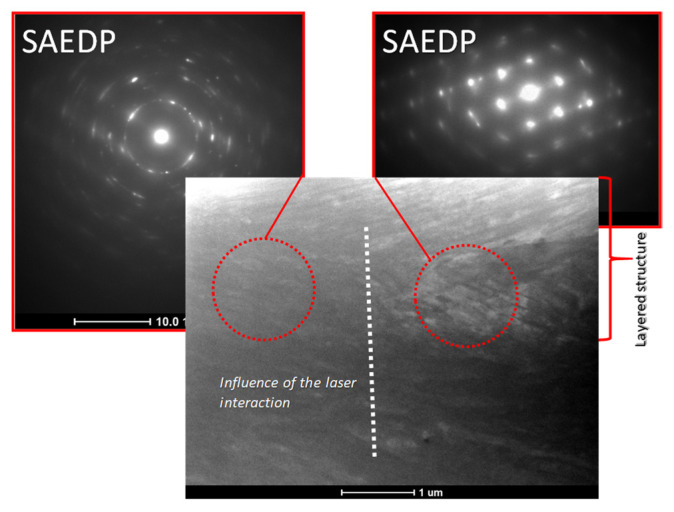
Microstructure TEM analysis of the substrate–layer interaction.

Studies of microstructural changes resulting from surface modification were performed using transmission electron microscopy. The aim of the study was to determine characteristic features such as dislocation density, grain and sub-grain size, distribution of precipitates, and their shape and size distribution. Observations of the microstructure in the dark field made it possible to determine the phase affiliation of a given precipitate and to characterize the ordering domains. Studies of this type were crucial, above all in determining the microstructural changes of areas at the substrate–coating interface (Figure 5). On the basis of the tests carried out, a significantly smaller grain size was found in the layer material compared to the substrate material.

### 3.3. Mechanical Evaluation of Thin-Film Materials

**Figure 6:**
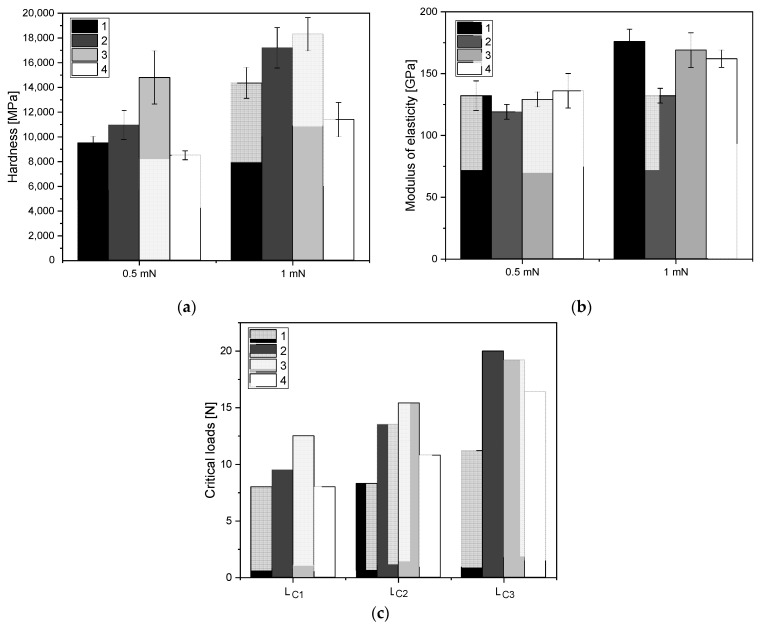
(**a**) Hardness values for 0.5 mN and 1mN; (**b**) Modulus of elasticity for 0.5 mN and 1 mN; (**c**) Critical loads: L_c1_ [N]—first cohesive cracks, L_c2_ [N]—first small area coating delamination, L_c3_ [N]—total coating delamination within the scratch track.

The results of hardness analyses of the tested layers are presented in Figure 6a for a loading force of 0.5 mN and 1 mN. While analyzing the hardness results, it was observed that the highest hardness, both for 0.5 mN and 1 mN loads, was obtained for coating 3 according to Table 1, manufactured using ALS method, at intermediate gas ratios C_2_H_2_:N_2_ 17.5:2.5. For coatings produced by the SPU method the highest hardness was obtained for the layer applied under 24:6:0 sccm gas flow conditions, respectively for Ar:C_2_H_2_:N_2_. 

The values of Young’s modulus are shown in Figure 6b for a load of 0.5 mN and 1 mN. The differences between the values for 0.5 mN were small and within the measurement scatter, staying within the range of 120–130 GPa. For indentation 1 mN, the highest value of Young’s modulus was characteristic for coating 1 manufactured using SPU method, at intermediate gas ratios Ar:C_2_H_2_ 24.0:6.0.

Figure 6c shows the force values corresponding to the critical loads L_c1_, L_c2_, L_c3_. The higher the value of the measured loads, the better the quality of the layer-substrate joint was. The critical load L_c1_ is determined for the value of the force at which the first cracks appear on the surface of the sample, which indicates the strength of the coating. The lowest value of the L_c1_, L_c2_, L_c3_ parameters is found in sample 1, where the loss of adhesion of the coating occurred at a value of 11.2 N. The best coating quality was observed for sample 3, where the first cracks appeared at a value of 12.5 N.

### 3.4. ATP Level and ADP/ATP Ratio

**Figure 7:**
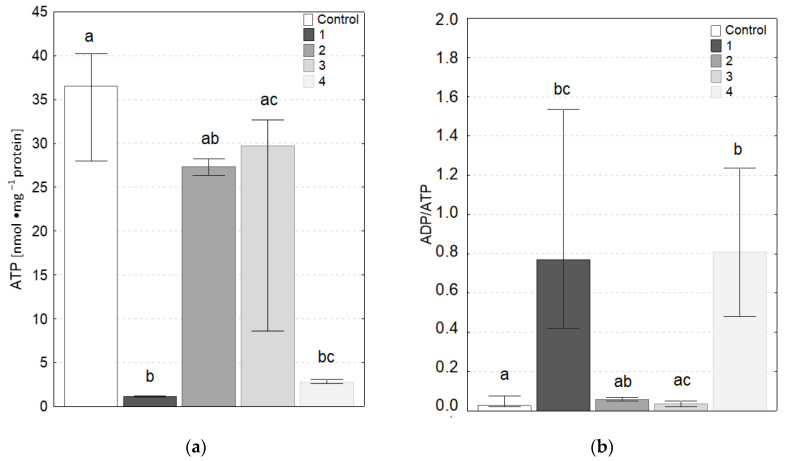
(**a**) Concentrations of ATP and (**b**) ADP/ATP ratio (Median ± quartile deviation; 25th and 75th percentiles) in fibroblasts from the control group and groups exposed to contact with selected coatings. The different letters (a, b, c) indicate statistically significant differences among groups (Kruskal–Wallis test, *p* < 0.05; N = 4–6).

The results of ATP concentration and ADP/ATP ratio are shown in Figure 7a,b, respectively. As can be seen, only cells in contact with coatings 2 and 3 (according to Table 1) were not statistically significantly different from the control. The contact of the cells with coatings 1 and 4 resulted in a decrease in ATP concentration and an increase in the ADP/ATP ratio, which may indicate an increase in degenerative changes (apoptosis, necrosis) in the cells. The measurements were repeated from 4 to 9 times. All samples were assessed in such way twice, as some measurements were eliminated due to too large data scatter. In principle, it can be assumed that these are indicative data.

### 3.5. Mechanical Evaluation of Thin-Film Materials

**Figure 8:**
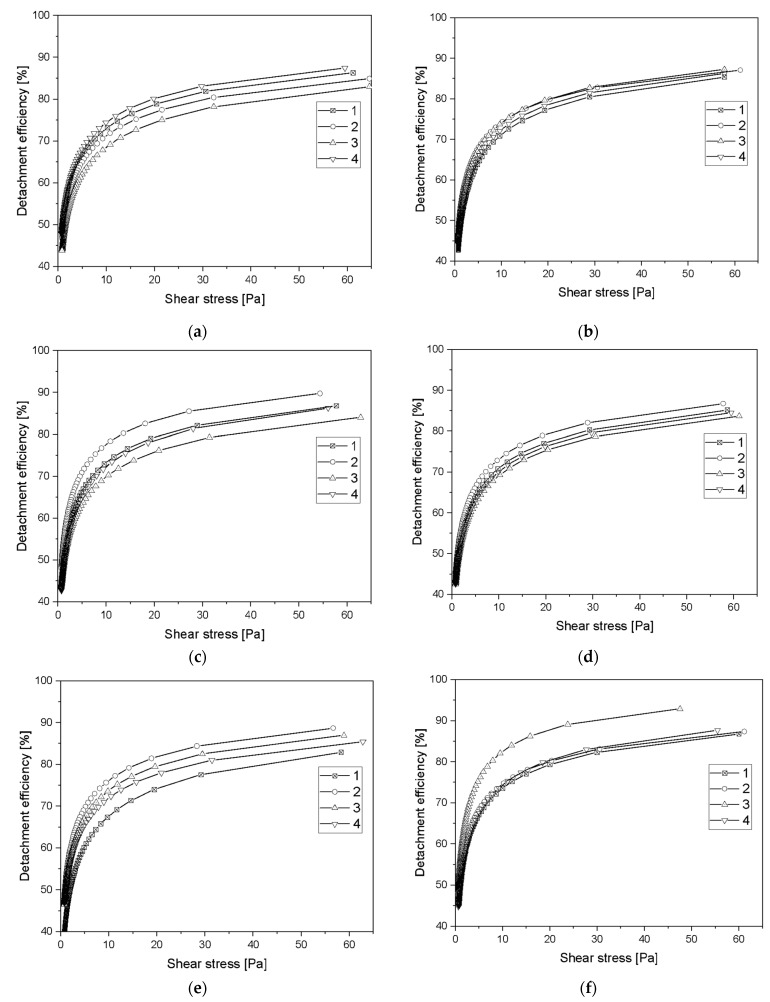

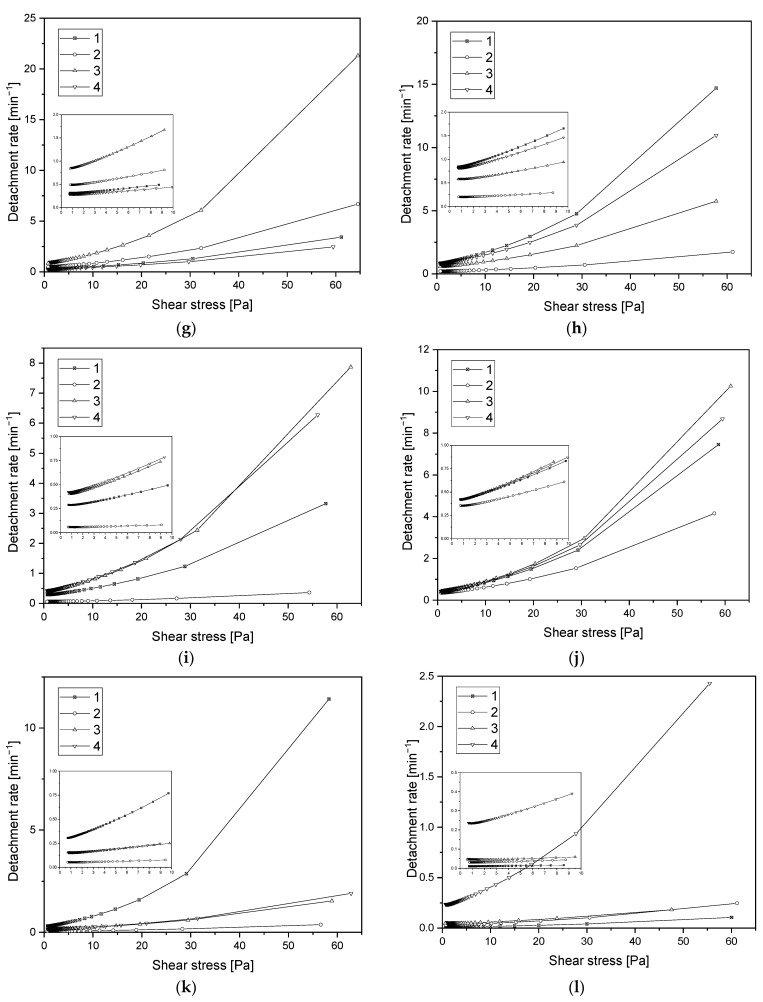
(**a**) Detachment efficiency of Red blood cells after 2.5 min flow; (**b**) Detachment efficiency of buffy coat after 2.5 min flow; (**c**) Detachment efficiency of Red blood cells after 5 min flow; (**d**) Detachment efficiency of buffy coat rate after 5 min flow; (**e**) Detachment efficiency of Red blood cells after 7.5 min flow; (**f**) Detachment efficiency of buffy coat after 7.5 min flow; (**g**) Detachment rate of Red blood cells after 2.5 min flow; (**h**) Detachment rate of buffy coat after 2.5 min flow; (**i**) Detachment rate of Red blood cells after 5 min flow; (**j**) Detachment rate of buffy coat rate after 5 min flow; (**k**) Detachment rate of Red blood cells after 7.5 min flow; (**l**) Detachment rate of buffy coat after 7.5 min flow.

The test based on the shear detachment assessment was focused on the determination of the detachment efficiency. In the case of the radial flow chamber, it had varying values of the shear stress depending on the distance from the leaching hole located in the central part. The flat surfaces of both the specimen and the disc caused the shear stress decrease with the increase of the radius “*r*”, meaning that at the edge of the sample, the shear stress was close to zero, and in the place where the inflow was given (sample center) its value was the highest. Detachment efficiency was described by the formula (3):

(3) $$E\left(\sigma \right)=\frac{1}{2}\left[1-Erf\left(\frac{\;l\;n\;\left(\frac{\sigma }{\sigma_{50\%}}\right)\;}{\sqrt{2\tilde{\sigma }}}\right)\;\right]$$

where *Erf* is the error function and *σ* is shear stress, $\tilde{\sigma }$ is the nondimensional variance, and $\sigma_{50\%}$ corresponds to the stress required to detach 50% of adherent cells. The detachment efficiency *E*(*σ*) is shown in Figure 8a–f. Detachment efficiency from the surface was about 75% for all tested times and samples. Only for sample 3, manufactured using ALS method, at intermediate gas ratios C_2_H_2_:N_2_ 17.5:2.5, was the detachment efficiency 83% (Table 2).

The detachment rate constant *k*(*σ*) characterizes the detachment kinetics at a given applied shear stress. It is the inverse of the average time needed to detach a cell submitted to a given hydrodynamic stress. The detachment rate is shown in Figure 8g–l. The flatter the detachment rate curve is, the weaker the cell–substrate interaction. As the stress increased, the percentage of cells detached from the substrate increased, which meant that the value of the detachment rate also increased.

Critical stress value determines the stress at which the probability of the cell being detached from the substrate is equivalent to its attachment. For sample 1, RBCs and the elution time of 7.5 min, the critical stress value was significantly higher than for the other samples (Table 2). This may have been caused by a process in which cells detach from the center of the sample and then re-adhere to the surface at less shear stress, which depends on the distance from the center of the sample where the inflow occurs. Spontaneous detachment rate is a rate at which cells detach from the surface when the system is not under shear stress. The lowest value of spontaneous detachment rate was also obtained for the sample 1 manufactured using SPU method, at intermediate gas ratios Ar:C_2_H_2_ 24.0:6.0 (Table 2).

### 3.6. Assessment of Hemocompatibility of Materials with Arterial Blood Flow

**Figure 9:**
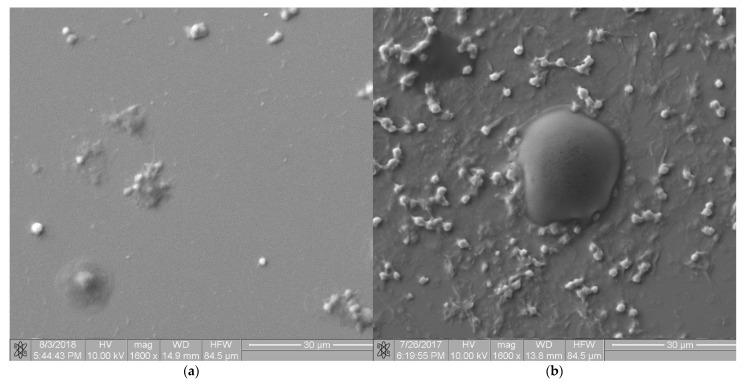
Blood–material interaction analysis using scanning electron microscopy (**a**) the occulted blood morphotypes on a-C:H surface; (**b**) the occulted blood morphotypes on a-C:H:N surface.

Microscopic images of the two extreme materials are shown in Figure 9. Figure 9a shows an image of the a-C:H surface, labeled “2”, according to Table 1. Figure 9b shows an image of the a-C:H:N surface, labeled “3”, according to Table 1.

**Figure 10:**
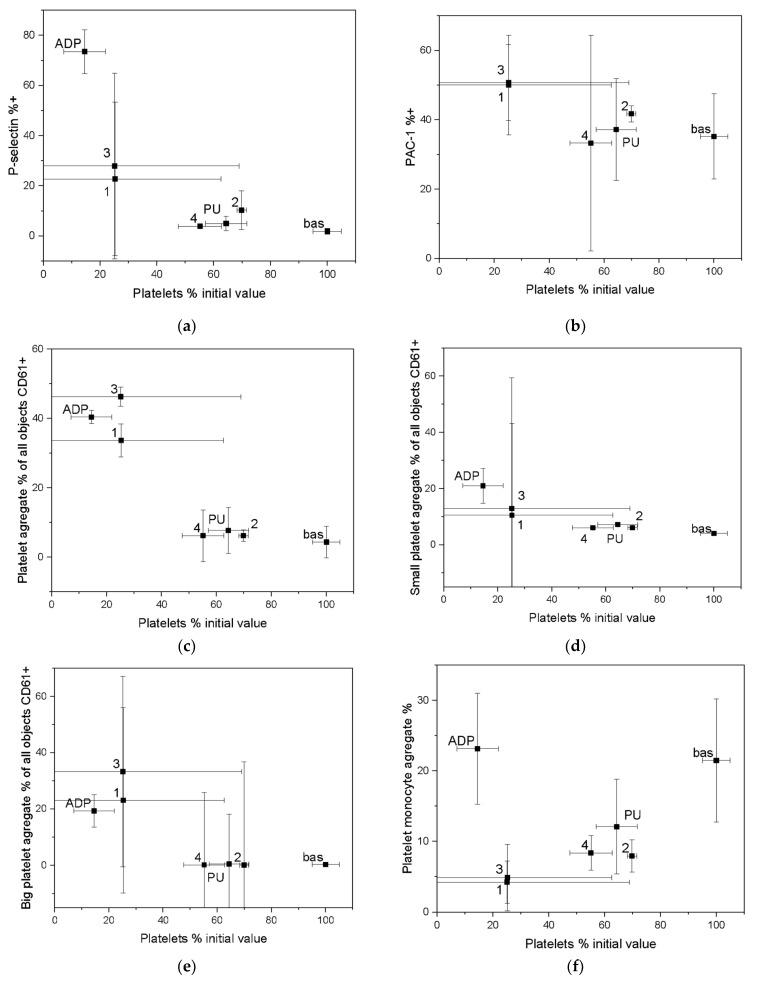

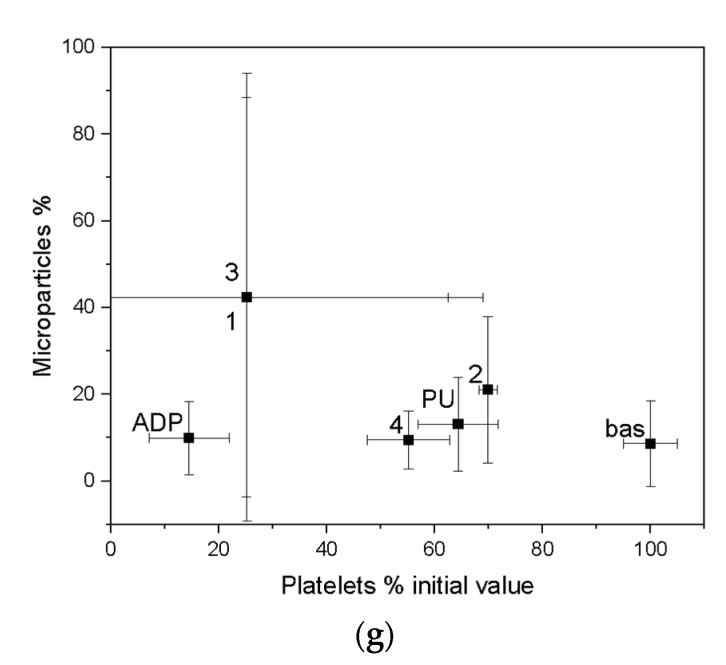
Blood–material interaction in the dynamic conditions. Bas—negative control, PU—reference substrate-medically used polyurethane, ADP—activated blood-positive control: (**a**) Activation of P-selectin receptor caused by direct contact with material under high shear condition; (**b**) Activation of platelet IIb/IIIa receptor caused by direct contact with the tested material under high shear condition; (**c**) All platelet aggregate caused by direct contact with material under high shear stress; (**d**) Small platelet aggregate caused by direct contact with material under high shear stress; (**e**) Large platelet aggregate caused by direct contact with material under high shear stress; (**f**) Platelet-monocyte aggregates created by direct contact with material under high shear condition; (**g**) Platelet-derived microparticles created by direct contact with artificial surface under shear stress condition.

Activation of a blood was measured as the percentage of platelets remaining in the blood after the shear test compared with the percentage of platelets expressing P-selectin (Figure 10a) and PAC-1 (Figure 10b). For the activation analysis of P-selectin, most coatings had little effect on activation processes, without consuming platelets that remained in the blood. The other IIb/IIIa receptor analyzed showed similar activation properties.

Throughout an activation process, platelet aggregation can occur by direct contact with an artificial surface under strong shear forces that are in direct contact with flowing blood. Excessive platelet aggregation is an uncontrolled physiological mechanism that regulates platelet aggregation. Under physiological conditions, platelet adhesion is activated by direct contact with an artificial surface to which platelets adhere. Activation of platelets releases substances that generate blood clotting.

The results of platelet aggregation after a shear test are compared with all CD 61 positive platelets (Figure 10c–e). A distinction can be made between small and large platelet aggregates. Small platelet aggregates are defined as two plates interconnected. Large platelet aggregates-more than two plaques joined together. Another biocompatibility comparison, taking into account the formation of platelet microparticles (MP) (Figure 10g), clearly demonstrated the lowest platelet consumption for coating 2 (according to Table 1) manufactured using SPU method, at intermediate gas ratios Ar:C_2_H_2_:N_2_ 24.0:6.0:0. This coating showed better biocompatibility parameters than clinically applied polyurethane. For coating 4, produced by the SPU (intermediate gas ratios Ar:C_2_H_2_ 28.2:1.8), the concentration of platelet microparticles slightly exceeded the human reference values while coating 3, manufactured using ALS method, showed the highest platelet activation under shear forces.

### 3.7. Assessment of Hemocompatibility of Materials with Arterial Blood Flow

**Figure 11:**
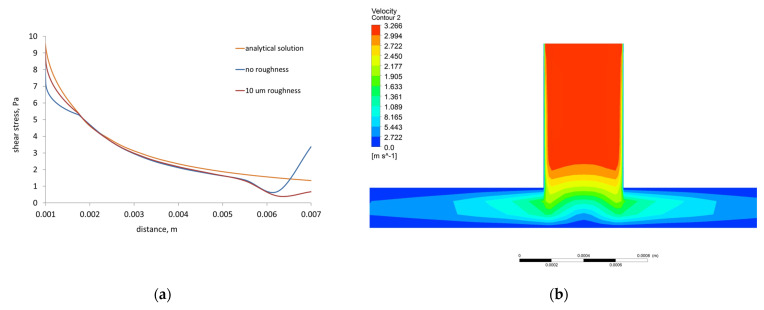

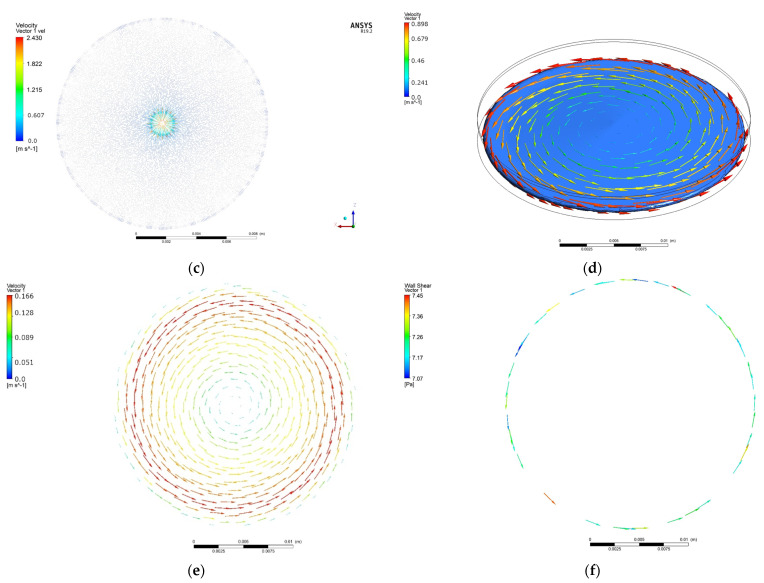
Results of 3D FVM radial flow test model: (**a**) Shear stress versus radius (distance from center of model) for models with roughness 10 μm and without roughness, and for analytical solution; (**b**) Distribution of velocity on the XY cross-section plane of model with roughness 10 μm; (**c**) Distribution of velocity on the XZ cross-section plane of model with roughness 10 μm. Results of 3D FVM Impact-R test model with roughness 10 μm: (**d**) Distributions of velocity vectors in whole model-bottom of cone, (**e**) Distributions of velocity vectors under rotating cone, (**f**) Distributions of shear stress vectors under rotating cone.

Referring to the phenomena related to the goal of the paper, several radial flow test models were simulated. The comparison of results reached by different models is shown in Figure 11a. The relation between shear stress and radii of model (distance from the center of the model) is similar for models with and without roughness. However analytical solution is more uniform and located above numerical shear-distance curves. A certain difference between the results obtained from the analytical solution and those calculated by the numerical model may be caused by the fact that the model gives results derived from the 3D solution. The analytical approach also does not consider influence of roughness and turbulence of flow. Thus, the distributions of velocity on the XY and XZ cross-section planes of 3D model of radial flow test are presented for 10 μm roughness (Figure 11 b,c).

The distribution of velocities on the cross-section plane *XY* (Figure 11b) shows that at the walls of the system, the velocities are lower due to the no-slip boundary condition. The velocity increases to approximately 3.266 m/s inside upper computational fluid domain due to the action of gravity along the *Y* axis and reaches 2.43 m/s on the bottom plane of the model. 

The numerical results of simulations computed under rotating cone for Impact-R test model with roughness 10 μm are presented as vector distributions of blood velocity and shear stress in Figure 11e,f.

In the whole 3D FVM Impact-R test model, the maximum value of blood velocity is 0.898 m/s (Figure 11d). The distributions of velocity vectors (Figure 9e) are uniform over the entire plane along the radius of the cylinder. On the analyzed plane, the velocity values are up to 0.166 m/s and the highest values are achieved near the inside wall of the cylinder. At the center of the analyzed cross-section plane there are very small velocity values that increase evenly along the radius of the cylinder toward its walls. In Figure 11e there are visible gradients of velocity at the cylinder wall, the maximum velocity values alternate with the minima, the latter being the closest to the cylinder wall.

In the case of vector distributions of wall shear stress on the cross-section plane directly under the cone, shear stress values are up to 7.45 Pa, the vectors are visible near the cylinder wall, and the computed shear stress values are the highest. There are visible gradients of shear stresses in Figure 11f at the perimeter of the analyzed cross-section plane. 

## 4. Discussion

On the basis of the research carried out, it is not possible to unequivocally conclude which material exhibits the best properties in terms of both mechanical and biological properties and, in particular, hemocompatibility. Therefore, at the selection stage, it was decided to select a so-called key property from the point of view of the purpose and possible future use. This key property was primarily hemocompatibility. The materials with the best hemocompatibility properties were selected with a view to have the least impact on blood activation and aggregation. The hemocompatibility properties under hydrodynamic conditions were compared to a clinically used material, i.e., polyurethane and a negative control. Only then were mechanical properties chosen for an evaluation and selection criterion. Unfortunately, no correlation was found between mechanical and biological properties (compare Table 3). The comparative analysis presented in Table 3 shows that the materials with the best biological properties are characterized by worse mechanical properties. The two extreme coatings (1 and 4) with the best and worst mechanical properties have the worst biological properties. Therefore, as a selection criterion, it was decided to choose biological properties, which indicate sample number 2. In turn, this material showed average properties mechanical in relation to other samples.

The ALS method significantly increased the hardness value as compared to the hardness of the coating produced by the SPU method. The results of nanoindentation and scratch tests exhibit that coatings 2 and 3 have the best properties among tested materials. From the functional point of view of substrate–coating systems, the most important parameter for biological applications is the strength of the coating–substrate interface. Fragments of the detached coating can be very dangerous when they transfer to the body, for example to bloodstream. Therefore, coatings with the highest critical load values show the greatest application potential. In addition, coatings 2 and 3 also have the highest hardness, which also indicates their greatest resistance to damage and wear during use. The introduction of hydrogen and nitrogen to carbon coatings significantly reduces the residual stress and nanohardness in the coatings compared to pure a-C coatings, and thus increases the strength of the connection with the substrate. The additionally introduced silicon nanoparticles can improve these properties, but only on the condition of the appropriate particle size (up to a few nanometers) and their separation in the microstructure of the coating. They stiffen the coating, which requires additional process of substrate hardening to prevent deformation leading to cracking of the coating.

The specific cellular response to mechanical stimuli depends on the strength of adhesion [68,69]. Pierrat et al. studied the mechanical force of adhesion and the dynamics of detachment of red blood cells from solid surfaces [68]. They used micropipette manipulation to create and then break the adhesive contact through a stepwise micromechanical procedure. However, due to the sensitivity of the cell membrane and the ease of disruption for contact-based assays, microfluidic platforms are more commonly used as an attractive tool to study cell mechano-sensitivity [70]. For example, microfluidic systems have been developed to study the effects of shear stress on the adhesion of erythrocytes [71], circulating tumor cells [72], leukocytes [73], and platelets to endothelium [74]. This device was used by Kucukal et al. to study red cell adhesion in microscale flows [75]. These works show the effect of shear forces on cell adhesion without considering the properties of the substrate. The present work uses a radial flow chamber, which was originally designed to analyze cell–material interactions under physiological conditions. The issue of cell adhesion force requires separate discussion. A high shear stress value should be considered as a high detachment force and good cell–surface adhesion.

The detachment efficiency of washing the cells from the surface for all samples is 75% for the stress of 60 Pa. The lower the stress value, the lower the detachment efficiency value. A flatter detachment velocity curve indicates the weaker the cell–substrate interaction. As the shear stress increases, the percentage of cells detached from the substrate increases, which means that the value of the detachment index also increases. The detachment rate allows for direct comparison of materials with each other, making it independent of the percentage of cells adhered to the surface. For the majority of samples, it is noticeable that the longer is the sample subjected to shear stress, the lower the cell detachment index is.

Fibroblasts were used as model cells in this study, due to the fact that they are key in modulating the response to implanted materials, such as long-term implant function or tissue regeneration [76]. Within each cell type there is a reasonably uniform quantity of ATP, and contact with coatings of potential implants may interfere process production of this high-energy phosphate compound and result in a reduction of the growth rate or viability cells [77]. Due to the fact that only two components of the adenylate pool, ATP and ADP, are involved in energy metabolism, ADP/ATP ratio is significant biomarker of metabolic conditions of cells. The high level of ATP point to proliferation processes, while high levels of ADP/ATP ratio indicate the extent of ATP inventory depletion and the progressive degenerative processes. Low values of ADP/ATP ratio (below 0.1), which were observed in cultured fibroblasts after contact with coatings a-C:H and a-C:H:N, show a balance between ATP production and ATP consumption in cells, therefore relatively low cytotoxicity of these materials. On the other hand, a decrease in ATP concentration and an increase in ADP/ATP ratio, compared with control, was observed in fibroblasts after an exposure to contact with coatings 1 and 4. Changes in the level of these parameters indicate a disturbance in energy metabolism of fibroblasts and strong cytotoxicity the mentioned coatings. Nevertheless, conclusions should be exercised with caution, because the type of cells used for testing is also a significant factor to be taken into account, as different cell lines prefer specific surface properties, thus displaying differences in their interaction with the coatings [78,79].

Similar to the material analyzed elsewhere, the coatings with the most favorable parameters for contact with blood were selected based on the activation and aggregation and the concentration of microparticles [80]. The obtained results provided important information with low likelihood of the microparticles formation under dynamic conditions, despite full activation using ADP. Under static conditions, the concentration of microparticles does not increase due to ADP blood activation, probably owing to the rapid aggregation of platelets. The tests exhibited a-C:H coatings with best hemocompatibility properties in direct contact with human blood. Taking into consideration the athrombogenic features of biomaterials, a lower cell adhesion force is desirable. In this case, the material itself may affect the platelets and coagulation system. However, low adhesion force makes it impossible to grow highly organized fibrin clots. a-C:H:F coatings were reported to possess great potential as coatings that can prevent thrombus formation on medical devices that come into contact with blood [81]. Horikawa et al. demonstrated that fluorine-incorporated amorphous carbon coatings control the initial thrombotic and inflammatory reactions of biomaterials by suppressing platelet adhesion and activation as well as neutrophil adhesion.

The comparative analysis of numerical simulations of the radial flow test and analytical approach show that in the 3D FVM model of the radial flow test, the shear stress decreases inversely proportional to the radius of the model, which is consistent with the analytical model [51]. However, the analytical solution does not consider roughness of surface, turbulence, or 3D solution. The developed 3D FVM radial flow test model reproduces the physical test well, and therefore qualitative and quantitative agreement between the experimental and numerical results without roughness is quite good. The shear stress values (below 10 Pa) obtained in the 3D FVM model of the radial flow test correspond to the calculated stresses for experimental samples. For these samples, experimental studies identified surface roughness on the level of micrometers. The 3D FVM radial flow test model considers the lowest roughness of the bottom wall equal to 10 μm.

In the case of no-slip boundary condition applied in the cone and plate test model, we only deal with shear stresses resulting from the interaction of cell adhesion molecules with a surface that has also been shown in the literature [82,83]. The calculated stress values were the stress values, at which there is a likelihood of spontaneous detachment of the cell from the material surface. The maximum values of wall shear stress obtained in literature for the cone and plate test model are from 0.3 Pa in [84] to 7 Pa in [85] for models without roughness. Therefore, in the present work the computed wall shear stress values involving roughness are in the range of literature data (to 7.45 Pa). Additionally, obtained tangential distributions of wall shear stress and velocity distributions increasing from the center of the model to its edge coincide with experimental and literature observations [84,85]. The cone and plate test model developed in the present study comprises not only roughness of the sample but also blood flow turbulence, thus the presented results show the output of more realistic blood–material interaction. The models with roughness are more sensitive to the number of computing time steps than models skipping roughness. Therefore, in the present paper, the model of cone and plate test computed for large number of time steps was applied.

## 5. Conclusions

The applied experimental research methods, both from the area of basic science and clinically applied ones, led to the following final conclusions. The aim of the study was achieved. Hemocompatible coatings on titanium ultimately dedicated to valve leaflet frame meshes were developed and applied. The designed materials were found to have properties confirmed in model tests for the planned application. A milestone was reached with the selection of optimal coating 2 composition for sealing. The selection was based on the selection of multilayer coatings in a comparative analysis of mechanical properties with polymer adhesion strength, ductility, and wear resistance. The correctness of the selected layers will be confirmed in the finished artificial valve model in biomechanical tests.

It was possible to formulate the main objective by achieving the individual product milestones that had been designated:

- The ALS coatings exhibit higher hardness values compared to the SPU coatings. Samples obtained by the ALS method also have a high Young’s modulus and are resistant to high critical loads.
- Hydrophilic properties influence cell adhesion. On the basis of the obtained test results, the hydrophilic properties of the developed materials were found, but without the risk of excessive activation of platelets.
- Based on the basic research test, based on radial flow chamber, the highest detachment efficiency obtained for ALS samples was observed. Samples obtained with the SPU method exhibit the lowest detachment rate and the highest critical stress value. Based on the observations obtained, these types of materials show a strong interaction with blood morphotic elements. It can be concluded on this basis that, from the point of view of the objectives of the work, these materials should probably be disqualified. However, the final decision was made on the basis of the clinical blood test. From the point of view of biomaterials engineering, however, this is not a parameter that must ultimately eliminate this type of material. The surface could strongly influence on the cell adhesion, but this does not at all indicate that the cells must be strongly activated.
- A decrease in ATP concentration and an increase in ADP/ATP ratio, compared with control, were observed in fibroblasts after an exposure to contact with coatings 1 and 4. Moreover, the coatings 2 and 3 showed no difference compared to the control.
- The observation resulting from the clinical whole-blood test showed the case mentioned in the previous application. The materials labelled 2, which showed strong interactions with blood morphotic elements in the primary test, showed at the same time the best hemocompatibility properties in whole blood tests.
- Materials marked as 2 (SPU) had the lowest platelet consumption, which ultimately ranked them as the best of the group of materials tested. Platelet-derived microparticles analyzed by Anexin V are another important indicator for material classification. Materials labelled with 2 showed the lowest risk of microparticle formation, which can be summarized as having the least impact on platelet destruction.
- For sample 4 (SPU), the concentration of platelet microparticles slightly exceeded the human reference values while coating 3, manufactured using ALS method, showed the highest platelet activation under shear forces.

The results of developed FVM models of the radial flow and cone and plate test lead to the following conclusions:

- The shear stress computed by the 3D FVM model of the radial flow test is in good qualitative and quantitative agreement with the shear stress calculated using analytical equation. The differences were caused by implementing roughness, turbulence, and 3D solution in the former.
- The cone and plate test model developed in the present study comprising roughness of the sample and blood flow turbulence, gives a better approximation of the blood–material interaction than the other attempts in this area.

## Figures and Tables

**Table 1:** Parameters of selected layers.

Sample	Material	Coating Thickness [nm]	Gas Flow [sccm]		Deposition Method
1	Si-a-C:H	15.0	24.0:6.0	Ar:C_2_H_2_	SPU (PULSED)
2	a-C:H	94.7	24.0:6.0:0	Ar:C_2_H_2_:N_2_	SPU (PULSED)
3	a-C:H:N	110.3	17.5:2.5	C_2_H_2_:N_2_	ALS
4	Si-a-C:H	124.3	28.2:1.8	Ar:C_2_H_2_	SPU (PULSED)

**Table 2:** Critical shear stress, spontaneous cell detachment rate, and detachment efficiency for erythrocytes and platelets.

Sample	Time [min]	Critical Tress [Pa]		Spontaneous Detachment Rate [min^−1^]		Detachment Efficiency [%]	
		Erythrocytes	Platelets	Erythrocytes	Platelets	Erythrocytes	Platelets
1	2.5	1.00	1.37	0.307	0.830	75.85	73.83
	5.0	1.10	1.36	0.279	0.415	75.85	73.71
	7.5	1.94	0.98	0.306	0.010	70.35	76.30
2	2.5	1.08	0.87	0.477	0.195	74.44	77.02
	5.0	0.79	1.11	0.056	0.344	79.72	75.77
	7.5	0.84	0.83	0.050	0.030	78.53	77.41
3	2.5	1.47	1.01	0.837	0.557	71.81	76.61
	5.0	1.34	1.53	0.394	0.414	72.95	72.10
	7.5	1.00	0.65	0.147	0.043	76.41	83.68
4	2.5	1.06	1.20	0.273	0.791	74.97	75.06
	5.0	1.30	1.45	0.413	0.415	74.71	72.93
	7.5	0.90	1.07	0.147	0.229	77.17	76.68

**Table 3:** Comparative analysis of tested materials.

Sample	Coating Thickness [nm]	Water Contact Angle	Hardness	Modulus of Elasticity	Critical Loads	Nucleotide Labeling	Critical Stress	Hemocompatibility
1	15	Average properties	Average properties	Average properties	Average properties	Average properties	Average properties	Average properties
2	94.7	Low value	The highest value among the SPU method	The lowest value	The best quality of the layer-substrate joint by the SPU method	The best properties	The lowest critical stress	The best properties
3	110.3	Average properties	The highest value among all methods	Average properties	The best quality of the layer-substrate joint among all methods	The best properties	The highest detachment efficiency	Average properties
4	124.3	The lowest value	Average properties	Average properties	Average properties	Average properties	Average properties	Average properties

## Data Availability

There was no need to use additional databases.

## Sample Availability

All samples are available from the authors.
